# Variations in the Quantity of Fatty Matter in the Human Lungs

**Published:** 1847-11

**Authors:** 


*Variations in the Quantity of Fatty Matter in the Human Lungs. —Relation to Jurisprudence.*—The following extract, taken from the Comptes Rendus of the 12th of July, from a paper read by M. Guillot before the Academy of Sciences, has some relation in subject to that given above, inasmuch as it shows the accumulation of fat in the lungs whenever their function is impaired.

M. Guillot first states the plan he has pursued in the examination and analysis of the lungs, on which his essay is based. He has first weighed the lungs in their moist state, the right and left, and whether healthy or diseased.

He has next separated the diseased from the healthy portions, and ascertained their respective weights. They have then been dried, in vacuo, at a temperature of 140°, and afterwards powdered, and then again dried. The powdered matter has next been treated with rectified ether, so as to remove all fatty matters. M. Guillot then informs us, that from his analysis he has found the amount of fatty matter contained in the pulmonary tissue to be more considerable in the fœtus before than after birth; that it diminishes from the moment the infant commences to breathe,

At the close of intra-uterine life, before the respiratory function is set up by birth, the proportion of fatty matter to the dried tissue of the lungs is from ten, twelve, fifteen, or eighteen to one hundred. But as soon as the air has entered the lungs, this proportion decreases to six parts to the hundred.

In all the affections of the chest, the consequence of which is to arrest permanently or transiently the respiratory act, in a greater or less extent, according to the extent of the lesion, the proportion of fatty matter increases in those parts rendered impermeable to the air. Under such circumstances, the rise in the quantity of such matters may be from fifteen to twenty, or thirty or forty, or even fifty parts to one hundred; whereas, in healthy portions of the same lungs, the proportion very rarely exceeds that of ten parts to the hundred. This fact hold good from the period of birth until advanced old age, in all cases where the lungs are obstructed by disease, or labour under phthisis. These organs, therefore, appear to undergo a fatty degeneration, which seems to be in relation with the absence of the contact of the air with the diseased parts.

The examination of lungs which are yet penetrated by air, notwithstanding extensive lesions, further supports this notion; for in such particular circumstances, the quantity of fatty matters extracted by means of ether is never equal to that afforded by portions of lungs in which air has ceased to enter during life.

According to the tables which the author has furnished, the greatest increase in the proportion of fat in the lungs takes place in phthisis, in which he has collected forty and even fifty-two parts in one hundred of desiccated pulmonary tissue. Here we may consider the fat to be derived both from the destruction of function of the lung, and also from the tubercular matter itself, which has long been observed to be mingled with much fat, so that it even seems but as a modification of that general pathological condition known as fatty degeneration.

Comparing lungs affected with pneumonia;—in those which are quite impermeable to air, the proportion of fat rises to fourteen, sixteen, and twenty-four parts in one hundred; whereas in those which are yet permeable, its proportion is as six to one hundred, in two out of three cases; in the third, as eleven to one hundred.

M. Guillot observes, these variations in the quantity of fat in the lungs, according as the process of respiration is free or interfered with,—rising in the latter case,—are interesting to physiologists, and seem to indicate that a part of the fatty matter entering the blood becomes consumed in its passage through the lungs by the act of respiration. In cases of pneumonia and phthisis, where the lungs become impermeable to air, this fat begins to accumulate until its quantity equals, and even surpasses, that observed in the liver, in which the quantity of fat is so appreciable and considerable.

From experiments I am now making, (says M. Guillot,) I am led to believe that the section of the pneumogastric nerves, and asphyxia, produce in animals phenomena analogous to those above pointed out.

Before quitting this matter, we would call our reader's attention to one point in the preceding paper which seems to us to have an important bearing on a much disputed point of medical jurisprudence—viz., *the distinction between respiration and inflation*, or insufflation, in the lungs, in cases of suspected infanticide. To determine this distinction is a great desideratum. Now, supposing the analyses of M. Guillot to be correct, can we not make use of them for the abovementioned purpose? He tells us, that as soon as the child has breathed, the quantity of fat in 100 parts of desiccated lungs diminishes but one-half at least, supposing the child to have arrived at the eighth or the ninth month of intra-uterine life. Thus, instead of the proportion of fatty matter being twelve, fifteen, or eighteen parts in 100, it is, by respiration, reduced to six parts. Now the question is, whether inflation will bring about this decrease of fatty matter as well as respiration. We should, *á priori*, think not; for this reduction of quantity is dependent on a vital action, and on certain chemical changes taking place in the blood under the influence of life. We can conceive how the pulmonary capillaries of the air-cells may become filled with blood by insufflation in a dead child, so as to resemble air-cells duly developed by respiration, but cannot understand how such a chemico-physiological change, as is the alteration of the proportion of fat in the lungs, can take place in such a manner, and after the extinction of life.

Be this as it may, the researches of M. Guillot are highly valuable in other respects, and are deserving of consideration and of repetition in this country; and if any relation or utility is perceived in them to forensic medicine, we shall have the merit of having directed the attention of the profession to them, and there will no doubt soon be inquirers in the same field to test the above researches in all their points of application.—*Ibid.*

